# Casticin inhibits interleukin-1β–induced ICAM-1 and MUC5AC expression by blocking NF-κB, PI3K-Akt, and MAPK signaling in human lung epithelial cells

**DOI:** 10.18632/oncotarget.20933

**Published:** 2017-09-15

**Authors:** Chian-Jiun Liou, Wen-Chung Huang

**Affiliations:** ^1^ Department of Nursing, Chang Gung University of Science and Technology, Taoyuan City, 33303, Taiwan; ^2^ Division of Allergy, Asthma, and Rheumatology, Department of Pediatrics, Chang Gung Memorial Hospital, Taoyuan City, 33303, Taiwan; ^3^ Graduate Institute of Health Industry Technology, Research Center for Food and Cosmetic Safety, and Research Center for Chinese Herbal Medicine, College of Human Ecology, Chang Gung University of Science and Technology, Taoyuan City, 33303, Taiwan

**Keywords:** casticin, ICAM-1, Interleukin-1β, MAPK, NF-κB

## Abstract

The compound casticin, isolated from *Vitex rotundifolia*, exerts anti-inflammatory effects and causes apoptosis of cancer cells. In this study, we explored the anti-inflammatory effects of casticin and modulation of cyclooxygenase (COX)-2, intercellular adhesion molecule 1 (ICAM-1), and mucin 5AC (MUC5AC) expression in interleukin-1β (IL-1β)–activated A549 human pulmonary epithelial cells. A549 cells were treated with various concentrations of casticin (5–20 μM), and an inflammatory response was triggered with interleukin (IL)-1β cytokines. Casticin decreased levels of IL-6, tumor necrosis factor α, and IL-8 and suppressed COX-2 expression and prostaglandin E2 production. It also reduced MUC5AC, proinflammatory cytokine, and chemokine gene expression and inhibited ICAM-1 expression for monocyte adhesion in IL-1β–stimulated A549 cells. In addition, casticin inhibited phosphorylation of Akt, phosphatidylinositol 3-kinase (PI3K), and mitogen-activated protein kinase (MAPK) and blocked nuclear transcription factor kappa-B (NF-κB) subunit p65 protein translocation into the nucleus. Co-culture of NF-κB, MAPK, and PI3K inhibitors with casticin also led to more significantly suppressed ICAM-1 expression in inflammatory A549 cells. These results provide evidence that casticin has an anti-inflammatory effect by blocking proinflammatory cytokine, chemokine, and ICAM-1 expression via suppression of the PI3K/Akt, NF-κB, and MAPK signaling pathways in IL-1β–stimulated inflammatory pulmonary epithelial cells.

## INTRODUCTION

The inflammatory response of airways is a physical danger signal and defense mechanism against microbe infection or injurious airborne particles [1]. Inflammatory pulmonary diseases include asthma, chronic obstructive pulmonary disease (COPD), and bacterial or viral pneumonia [2, 3]. These reactive airway diseases can lead to sputum production, coughing, and wheezing, and the airway of patients also may be stimulated to constrict, with excessive mucus secretion leading to dyspnea or suffocation and death [4]. With a bacterial or viral infection, activation of macrophages and T cells of airways occurs, and more interleukin (IL)-1β can be detected in inflammatory diseases of the airways [5]. It had been previously found that influenza A virus major targeted epithelial cells of airway and lung to induce airway inflammatory and caused the damage of lung tissue. Influenza A virus could activate innate immune to secret IL-1β for caused airways damage [6]. Recent studies have also found that lung epithelial cells can release excessive proinflammatory cytokines, including IL-1β and tumor necrosis factor α (TNF-α), triggering a severe immune-inflammatory response in asthmatic patients [7]. In IL-1 receptor knockout mice, neutrophil infiltration of the lungs is significantly decreased in lipopolysaccharide (LPS)-induced lung injury, and these animals show a suppressed airway hyperresponse compared with wild-type asthmatic mice [8]. Anti-inflammatories, such as non-steroidal anti-inflammatory drugs, could attenuate airway inflammatory disease [9, 10]. Cyclooxygenase 2 (COX-2) breaks down arachidonic acid to produce prostaglandins, which can increase inflammation, fever, and pain [9]. Non-steroidal anti-inflammatory drugs decrease COX-2 activity and reduce inflammation and fever in these patients [11, 12]. Celecoxib is a COX-2 inhibitor that ameliorates newborn hyperoxic lung injury in mice and decreases levels of prostaglandin E2 (PGE_2_) to suppress lung inflammation in asthma patients [13, 14]. Hence, blocking IL-1β levels could ameliorate the lung inflammatory response in pulmonary disease patients.

A previous study found that inflammatory lung epithelial cells express the IL-1 receptor [15]. IL-1β binds the IL-1 receptor, inducing activity of inflammatory signaling pathways in lung epithelial cells, leading to the release of proinflammatory cytokines, chemokines, and inflammatory mediators. Nuclear transcription factor kappa-B (NF-κB) is an important inflammation signaling molecule in IL-1β–stimulated lung epithelial cells [16]. When inflammatory molecules stimulate IκB phosphorylation, NF-κB subunits p65 and p50 are also translocated into the nucleus to trigger expression of inflammation-associated cytokines and mediator genes [17, 18]. IL-1β–stimulated lung epithelial cells also activate the mitogen-activated protein kinase (MAPK) and phosphatidylinositol 3-kinase (PI3K) signaling pathways to exacerbate the inflammatory response [19]. In turn, inflammatory lung epithelial cells release more chemokines to attract leukocytes, and the surface of lung epithelial cells expresses intercellular adhesion molecule 1 (ICAM-1) to attract macrophage, neutrophil, or eosinophil infiltration into the lung tissue [20, 21]. Thus, reducing ICAM-1 expression in lung epithelial cells also might ameliorate respiratory inflammatory diseases.

Recent studies found that some bacterial infection could increase IL-1β production to activate NLRP3 inflammasome for caused community-acquired pneumonia [22]. *Streptococcus pneumoniae* could destroy the effect of plasma membrane of lung epithelial cells for interfered K^+^ efflux by activation of the NLRP3 inflammasome [23]. Activated immune cells released IL-1β to cause inflammatory response and increase the body temperature to attenuate microbial activity. The blood and sputum of asthma and COPD patients found that IL-1β gene expression was significantly higher than healthy people [24]. Moreover, IL-1β-related signaling pathway also significantlly activated in the lung of asthma and COPD patients [25]. Hence, to reduce the IL-1β levels and inflammation of local lungs could improve the incidence of asthma and COPD. Furthermore, obese mice treated with IL-1β antagonist could attenuate airway hyperresponsiveness and inflammtion of lung [26]. Breast and lung cancer patients also found that could detect more high IL-1β levels compared to health adult. Clinical treatment reduced IL-1β levels of lung would have an opportunity to improve inflammation and the development of lung cancer [27].

*Vitex rotundifolia* L. grows in the countryside of China and Taiwan, and its fruit has been used to treat inflammation, gastroenteritis, and headaches in traditional Chinese medicine [28]. Casticin, isolated from *V. rotundifolia*, has been reported to have anti-tumor effects *in vitro* [29]. Previously, we found that casticin could suppress the inflammatory effect by blocking the NF-κB and MAPK pathways in LPS-induced RAW264.7 macrophage cells [30]. Casticin also decreases the levels of eotaxin and reduces eosinophil migration in IL-1β–stimulated A549 human lung epithelial cells [28].

In this study, we evaluated the anti-inflammatory effect of casticin and explored the mechanism of involvement of the NF-κB, PI3k/Akt, and MAPK signaling pathways in IL-1β–stimulated A549 cells.

## RESULTS

### Casticin inhibited proinflammatory cytokine and chemokine production in IL-1β–stimulated A549 cells

The cytotoxicity of casticin in A549 and H460 cells was determined by MTT assay.

**Figure 1:**
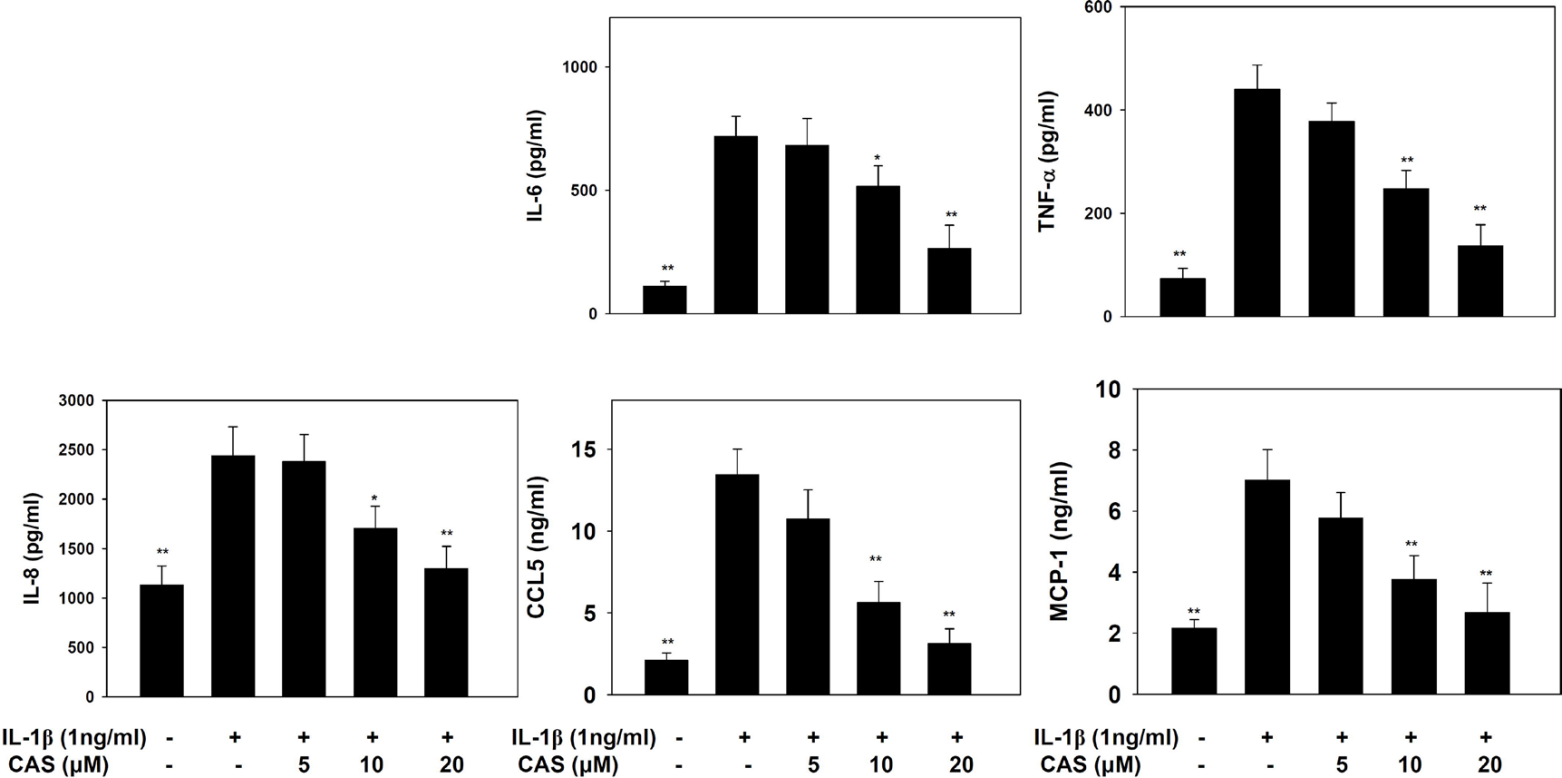
The effects of casticin (CAS) on IL-1β–induced production of IL-6, IL-8, TNF-α, CCL5, and MCP-1 A549 cells (10^6^ cells/well) were pretreated with CAS for 1 h and then stimulated with IL-1β (1 ng/ml) for 24 h. The presented data are mean ± SEM; ^*^p < 0.05, ^**^p < 0.01, compared with the IL-1β–treated group.

**Figure 2:**
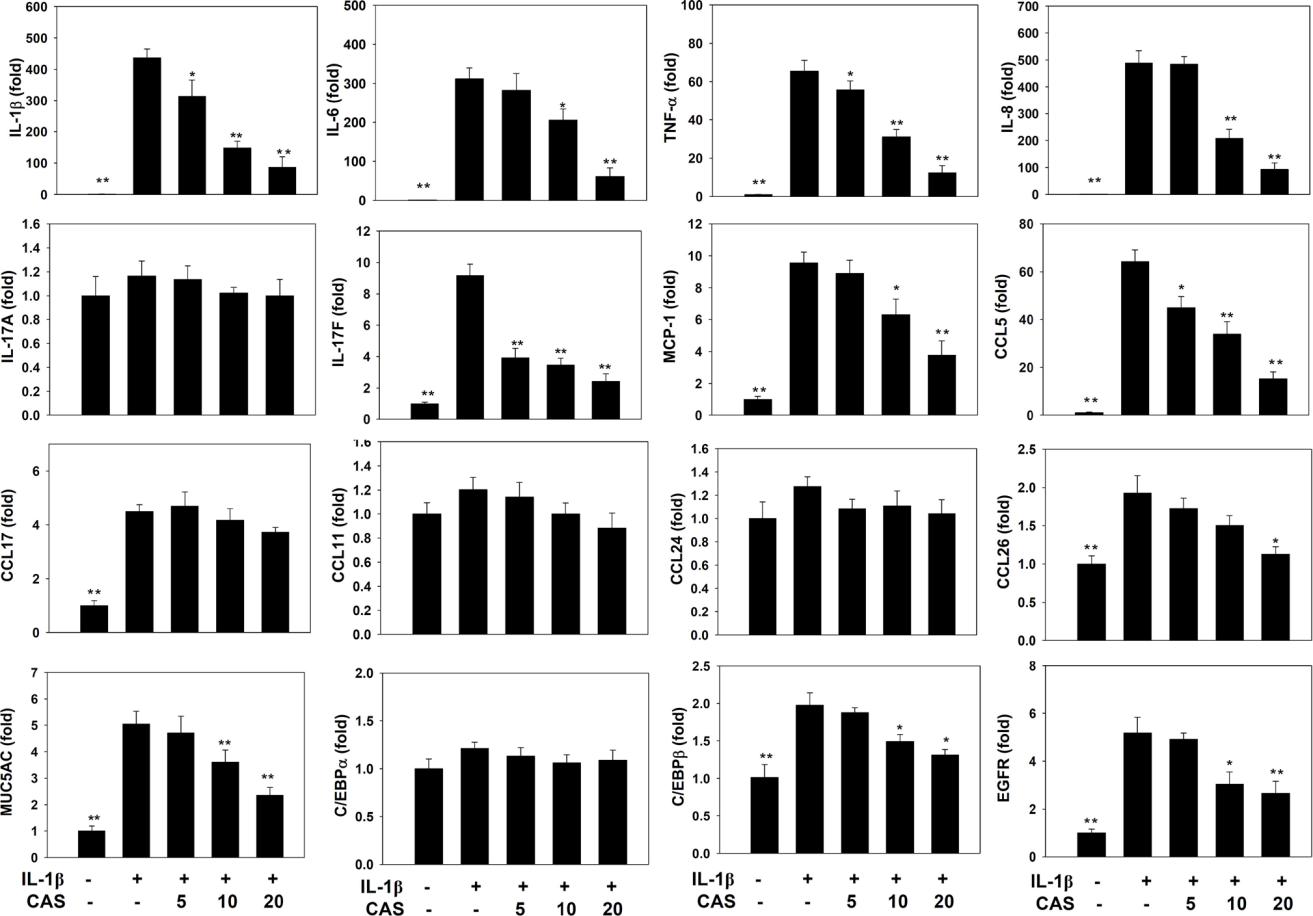
Effects of casticin (CAS) on IL-1β–induced gene expression A549 cells (10^6^ cells/ml) were pretreated with CAS for 1 h and then stimulated with IL-1β (1 ng/ml) for 4 h to assay gene expression levels, determined using real-time RT-PCR. The presented data are mean±SEM; ^*^p < 0.05, ^**^p < 0.01, compared with the IL-1β–treated group.

Casticin did not significantly affect cell cytotoxicity at doses ≤ 20 μM, and all experiments used casticin from 5–20 μM (Supplementary Figure 1A). Next, cells were treated with different doses of IL-1β (0.5–5 ng/ml) for 24 h. A549 cells could significantly increase the levels of IL-6 and IL-8 in a dose-dependent manner compared with untreated cells (Supplementary Figure 1B, 1C). We found that IL-1β-stimulated H460 did not significantly increase IL-6 and IL-8 productions. Furthermore, casticin could decrease the levels of IL-6 and IL-8 without IL-1β–stimulated H460 cells (Supplementary Figure 1D, 1E). Hence, A549 cells were used to evalute the anti-inflammatory effects of casticin. Casticin had a dose-dependent inhibitory effect on levels of IL-6, TNF-α, IL-8 (IL-6: 5 μM casticin, 681.86 ± 109.45 pg/ml, *p*=0.34; 10 μM casticin, 516.05 ± 83.03 pg/ml, *p* < 0.05; 20 μM casticin, 263.91 ± 54.85 pg/ml, *p* < 0.01; vs. IL-1β alone, 717.21 ± 83.08 pg/ml) (TNF-α: 5 μM casticin, 377.92 ± 35.90 pg/ml, *p* = 0.22; 10 μM casticin, 247.29 ± 35.86 pg/ml, *p* < 0.01; 20 μM casticin, 136.70 ± 40.97 pg/ml, *p* < 0.01; vs. IL-1β alone, 439.59 ± 47.50 pg/ml), and casticin also could decrease the levels of IL-8, CCL5, and MCP-1 in IL-1β–stimulated A549 cells (Figure 1). We also evaluated the gene expression of proinflammatory cytokines and chemokines by real-time PCR and found that casticin significantly suppressed IL-1β, IL-6, TNF-α, IL-8, CCL5, MCP-1, IL-17F, and CCL26 (Figure 2). However, it did not significantly modulate IL-17A, CCL11, CCL17, or CCL24 gene expression. Additionally, casticin inhibited MUC5AC, C/EBPβ, and epidermal growth factor receptor (EGFR), but C/EBPα did not show decreased gene expression in A549 cells.

### Casticin suppressed COX-2 expression in IL-1β–stimulated A549 cells

**Figure 3:**
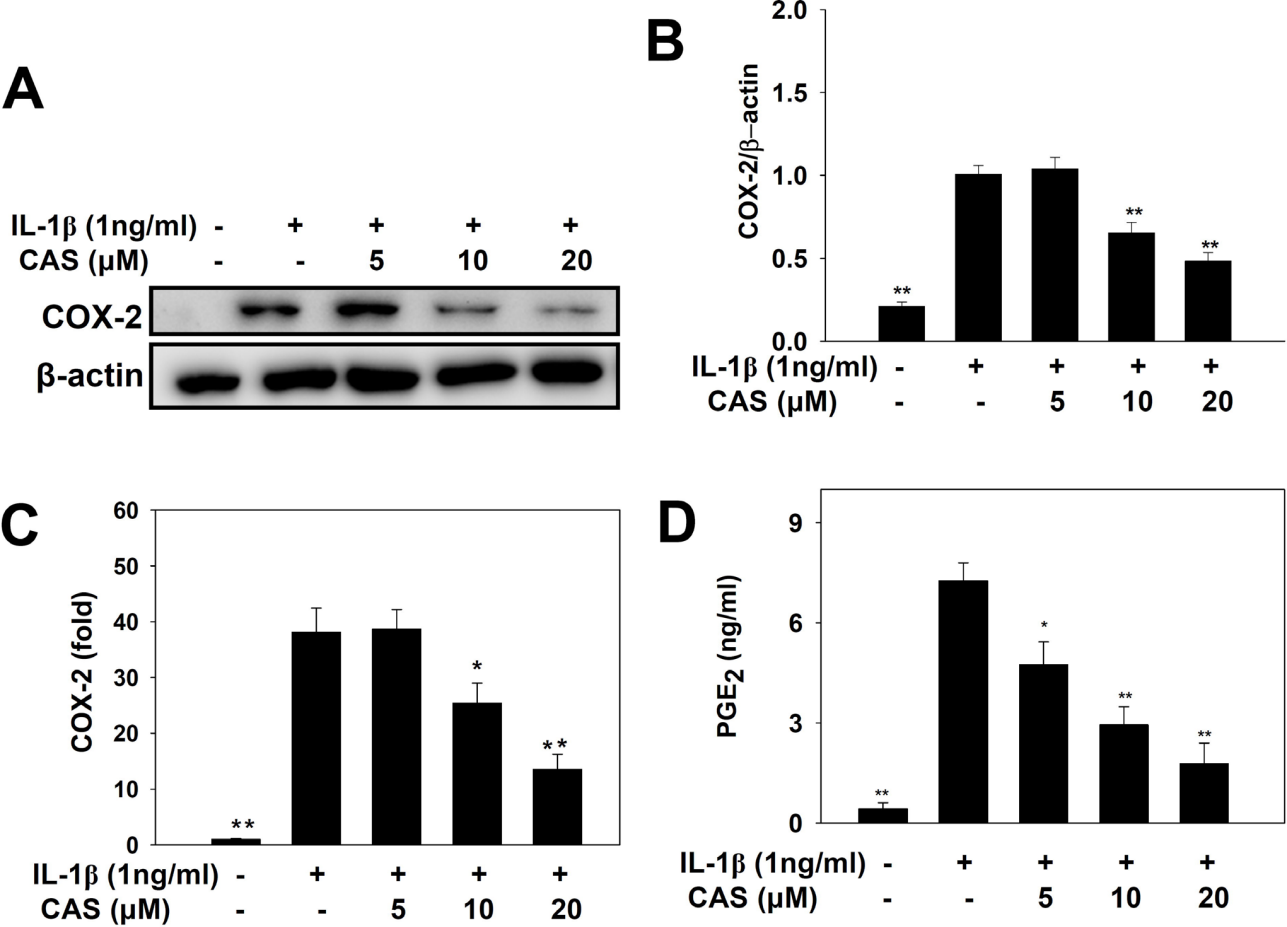
Effects of casticin (CAS) on IL-1β–induced production of COX-2 and PGE_2_ A549 cells (10^6^ cells/ml) were pretreated with CAS for 1 h and then stimulated with IL-1β (1 ng/ml) for 24 h. COX-2 proteins were detected using β-actin as an internal control (**A**), and COX-2 protein expressions were measured relative to the expression of β-actin (internal control) (**B**). COX-2 gene expression was measured by real-time PCR (**C**), and levels of PGE_2_ were evaluated by ELISA (**D**). Data are presented as mean ± SEM; ^*^p < 0.05, ^**^p < 0.01, compared with the IL-1β–treated group.

When A549 cells were treated with various concentrations of casticin and then stimulated with IL-1β, casticin significantly suppressed COX-2 protein expression compared with IL-1β–stimulated cells (Figure 3A, 3B). Real-time PCR analysis revealed that casticin also decreased COX-2 gene expression in a concentration-dependent manner (Figure 3C). In addition, we found that casticin significantly reduced the level of PGE_2_ (5 μM casticin, 4.74 ± 0.68 ng/ml, *p* < 0.05; 10 μM casticin, 2.94 ± 0.55 ng/ml, *p* < 0.01; 20 μM casticin, 1.77 ± 0.62 ng/ml, *p* < 0.01; vs. IL-1β alone, 7.25 ± 0.53 ng/ml) (Figure 3D).

### Casticin suppressed ICAM-1 expression in A549 cells

**Figure 4:**
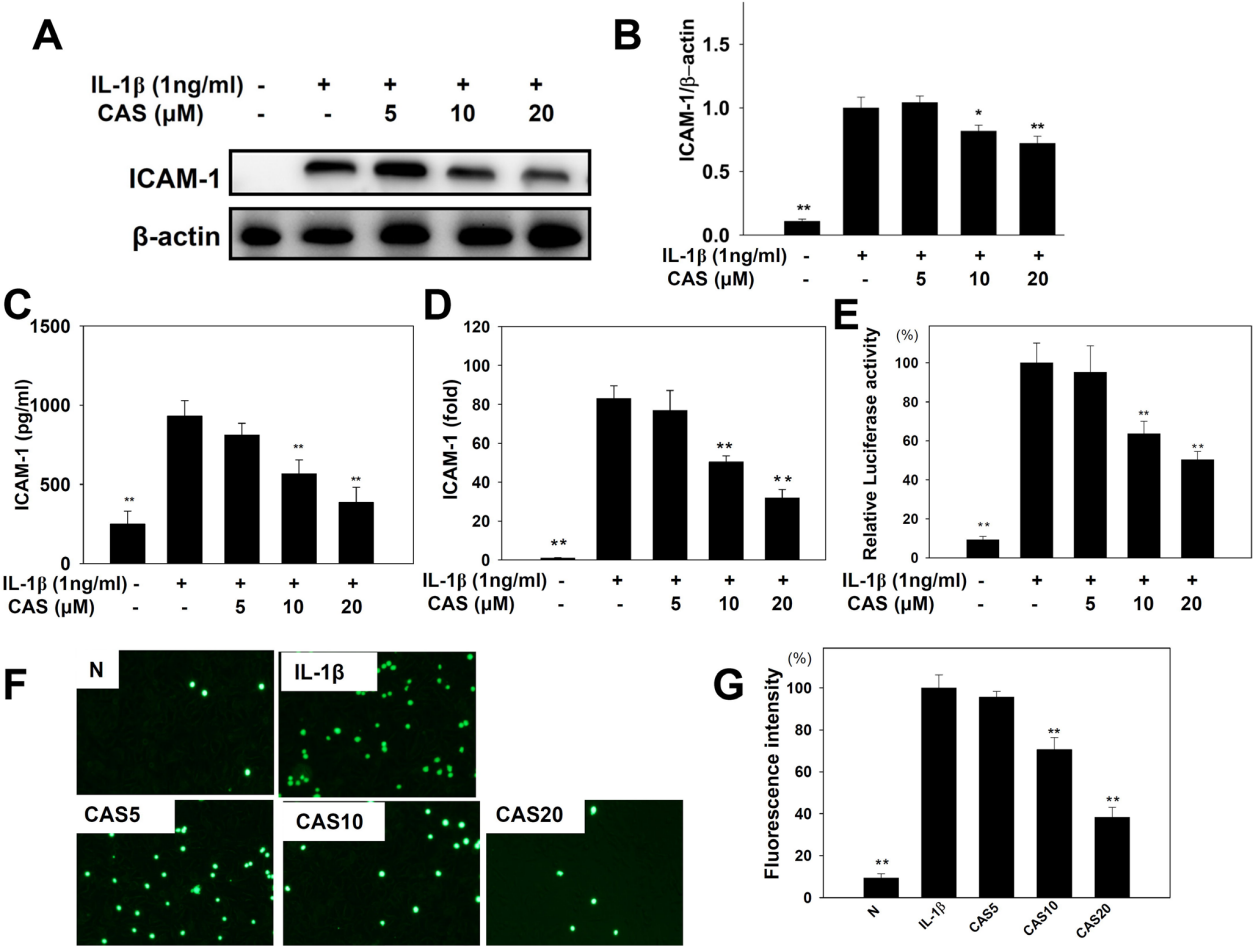
Effects of casticin (CAS) on IL-1β–induced production of ICAM-1 A549 cells (10^6^ cells/ml) were pretreated with CAS for 1 h and then stimulated with IL-1β (1 ng/ml) for 24 h to assay ICAM-1 protein expression by western blot (**A**), and ICAM-1 protein expressions were measured relative to the expression of β-actin (internal control) (**B**). ICAM-1 of the culture medium by ELISA (**C**). ICAM-1 gene expression levels, determined using real-time RT-PCR (**D**), and promoter assay were used to evaluate ICAM-1 promoter activity (**E**). THP-1 cells were labeled with calcein AM and co-cultured with A549 cells, followed by observation using fluorescence microscopy (**F**). Fluorescence intensity of THP-1 cell adhesion to A549 cells (**G**). The presented data are mean ± SEM; ^*^p < 0.05, ^**^p < 0.01, compared with the IL-1β–treated group.

**Figure 5:**
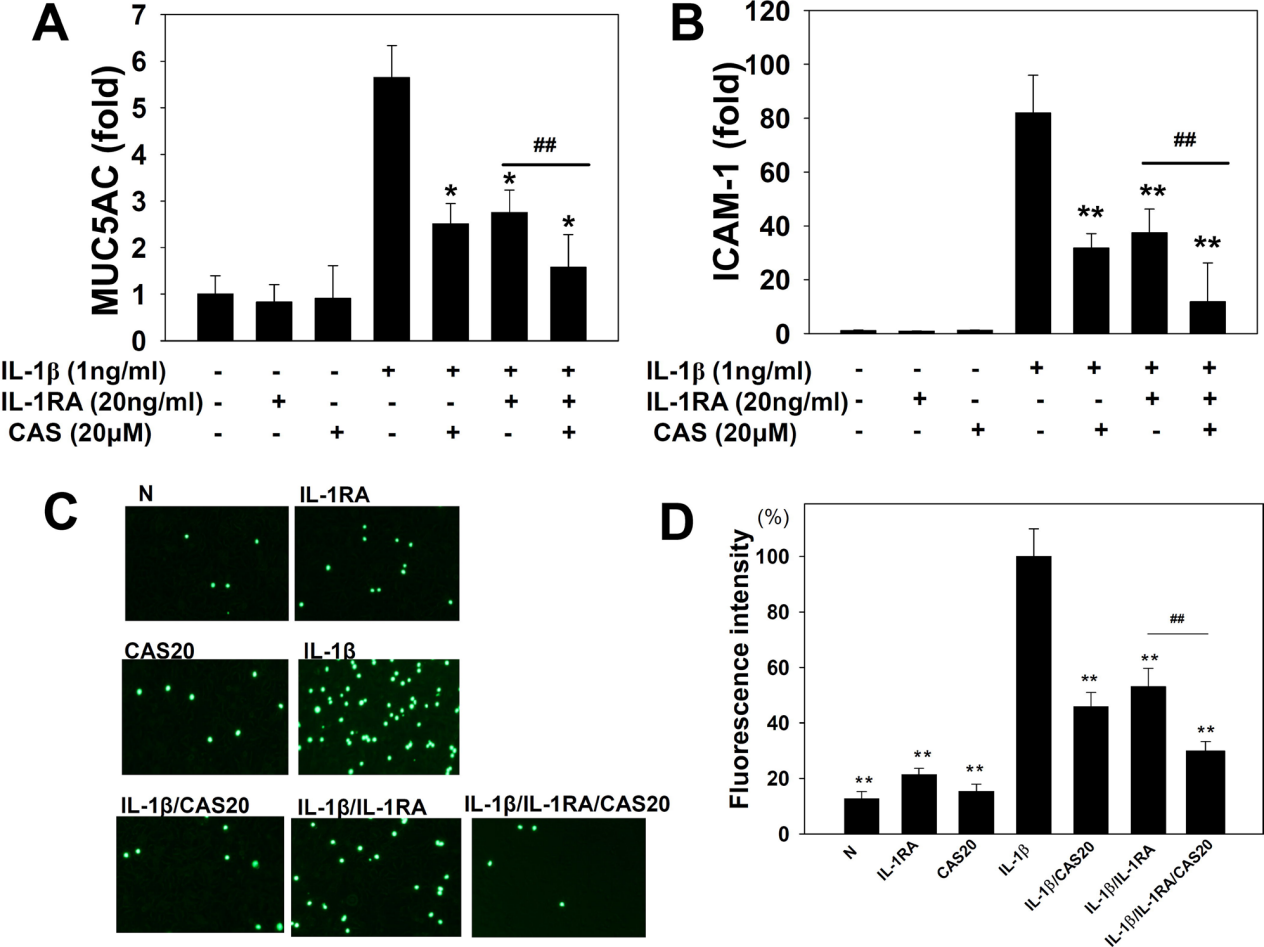
Effects of casticin (CAS) or interleukin-1 receptor antagonist (IL-1RA) on the IL-1β–induced production of ICAM-1 and MUC5AC A549 cells (10^6^ cells/ml) were pretreated with casticin (CAS) or (20 ng/ml) IL-1RA for 1 h and then stimulated with IL-1β (1 ng/ml) for 4 h to assay MUC5AC (**A**) and ICAM-1 (**B**) gene expression, determined using real-time RT-PCR. THP-1 cells were labeled with calcein AM and co-cultured with A549 cells, followed by observation using fluorescence microscopy (**C**). Fluorescence intensity of THP-1 cell adhesion to A549 cells (**D**). The presented data are mean±SEM; ^*^p < 0.05, ^**^p < 0.01, compared with the IL-1β–treated group. ^#^*p* < 0.05, ^#^*p* < 0.01, compared with the IL-1β/IL-1RA group.

The ICAM-1 protein assay showed that casticin significantly reduced ICAM-1 expression (Figure 4A, 4B) and suppressed soluble ICAM-1 release into culture medium compared with IL-1β–stimulated A549 cells (Figure 4C). Real-time PCR investigation showed that casticin significantly inhibited ICAM-1 gene expression in A549 cells (Figure 4D). Casticin also significantly suppressed luciferase activity by blocking ICAM-1 promoter activity (Figure 4E). We additionally evaluated whether casticin could suppress the ability of monocyte THP-1 cells to adhere to IL-1β–stimulated A549 cells. Pretreatment with casticin decreased adhesion of THP-1 cells to A549 cells (Figure 5F, 5G).

### Casticin suppressed ICAM-1 and Muc5AC expression in A549 cells treated with an IL-1 receptor antagonist

IL-1β–stimulated A549 cells showed increased IL-1β gene expression (Figure 2), suggesting that these cells have IL-1 receptor activity and expression. Previously, it was shown that IL-1β could stimulate IL-1 expression and that the IL-1 receptor antagonist (IL-1RA) could block the IL-1β signaling pathway [31]. Thus, IL-1RA would be expected to lead to decreased ICAM-1 and MUC5AC gene expression in IL-1β–stimulated A549 cells, as we found (Figure 5A, 5B). We also found that casticin could more significantly decrease ICAM-1 and MUC5AC gene expression when IL-1β–stimulated A549 cells were treated with IL-1RA. In addition, pretreatment with casticin even further suppressed the adhesion of THP-1 cells to IL-1β/IL-1RA–treated A549 cells (Figure 5C, 5D).

### Inhibition by casticin of inflammatory signaling pathways in IL-1β–activated human lung epithelial cells

**Figure 6:**
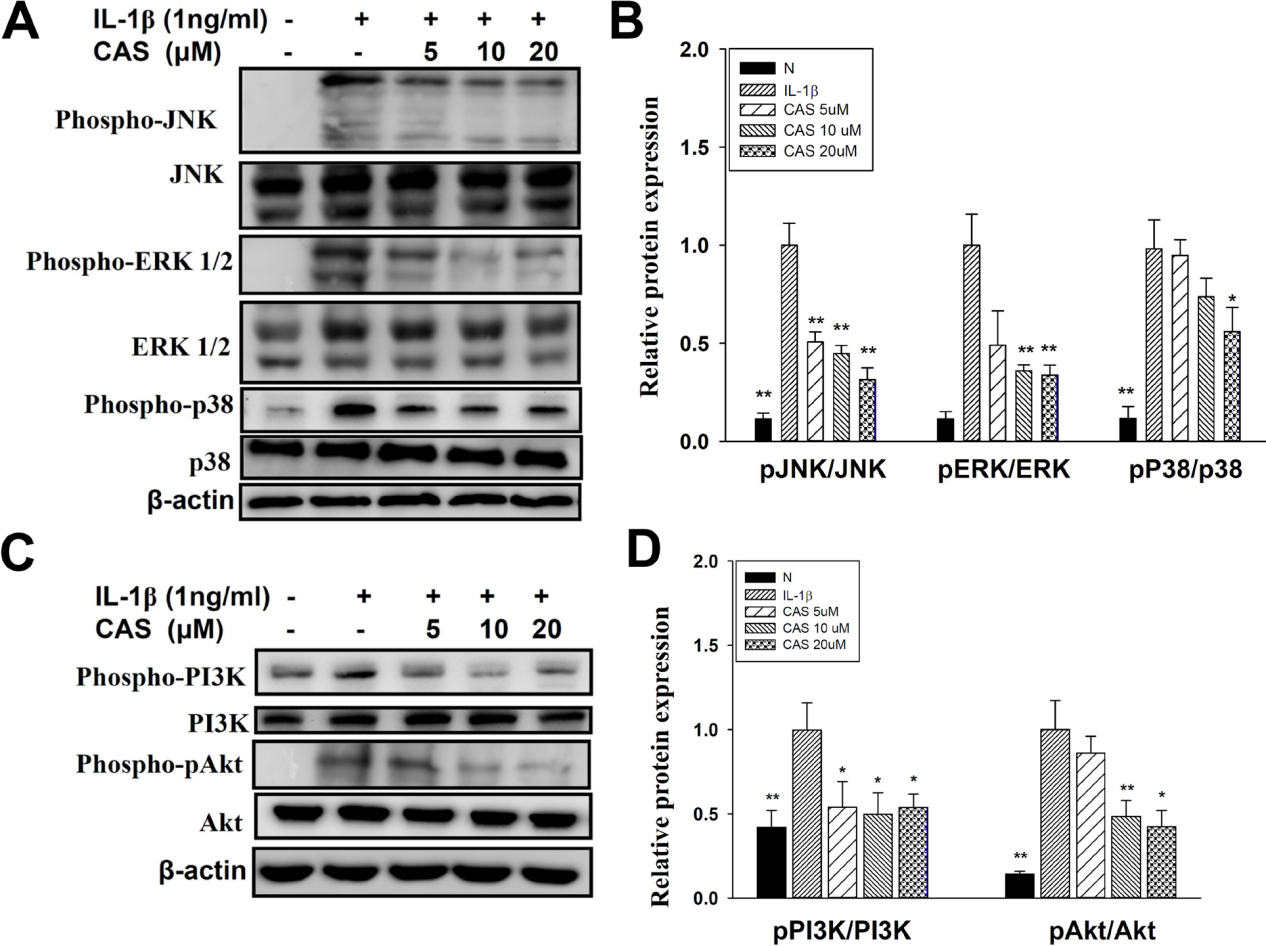
Effect of casticin (CAS) on IL-1β–induced activation of PI3K-Akt, and MAPK signaling in A549 cells A549 cells were pretreated with CAS for 1 h and then incubated with IL-1β (1 ng/ml) for 30 min. Phospho-specific proteins were detected using western blot, and total MAPK (**A**, **B**) and PI3K/Akt (**C**, **D**) levels were used as internal controls. The presented data are mean ± SEM; ^*^p < 0.05, ^**^p < 0.01, compared with the IL-1β–treated group.

**Figure 7:**
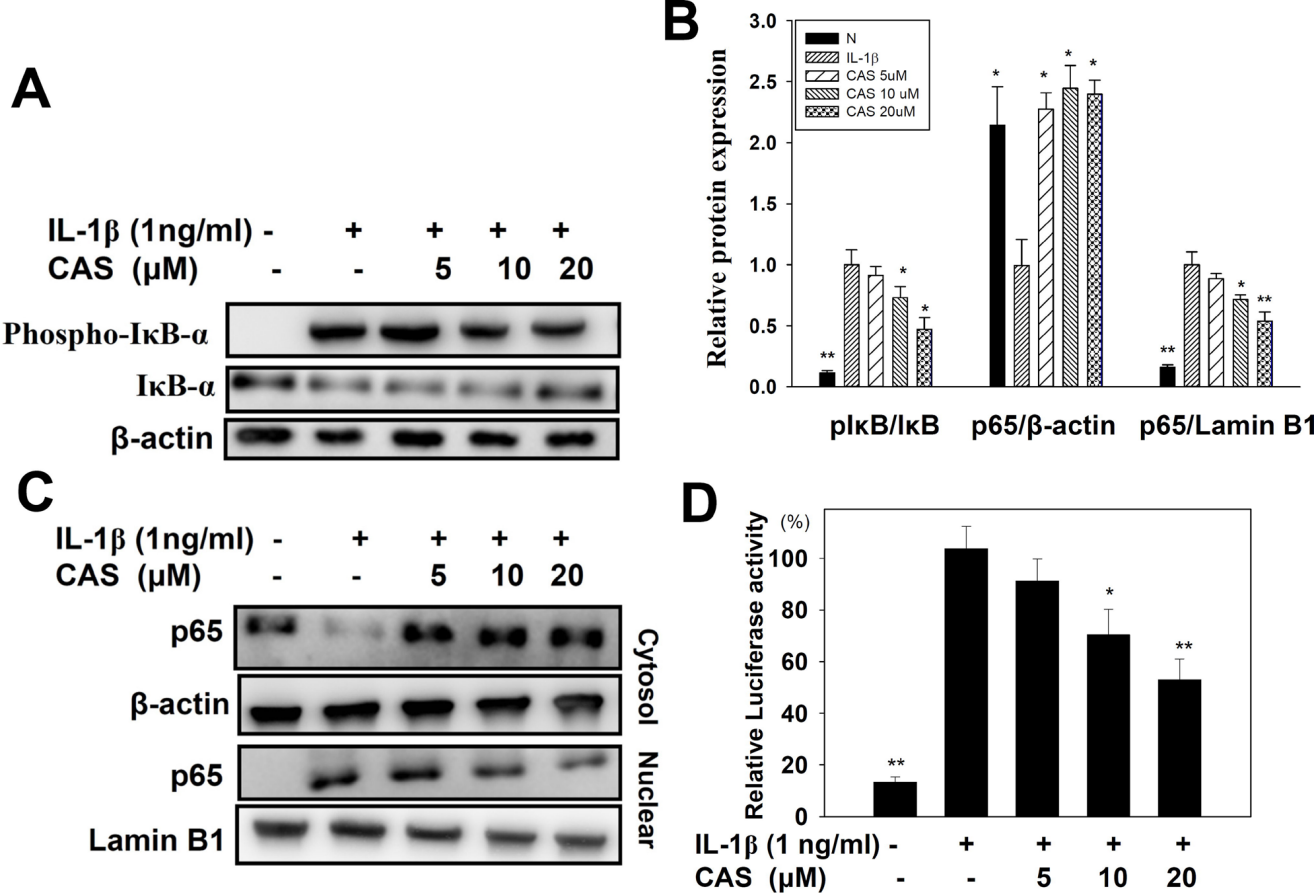
Effect of casticin (CAS) on IL-1β–induced activation of NF-κB signaling in A549 cells A549 cells were pretreated with CAS for 1 h and then incubated with IL-1β (1 ng/ml) for 30 min. Phospho-specific proteins were detected using western blot, and total IκB-α (**A**, **B**) levels were used as internal controls. For the nuclear translocation of NF-κB, cells were pretreated with CAS for 1 h and then incubated with IL-1β (1 ng/ml) for 1 h (B, **C**). The internal controls were Lamin B1 in the nucleus and β-actin in the cytosol. A promoter assay was used to evaluate NF-κB promoter activity (**D**). The presented data are mean ± SEM; ^*^p < 0.05, ^**^p < 0.01, compared with the IL-1β–treated group.

**Figure 8:**
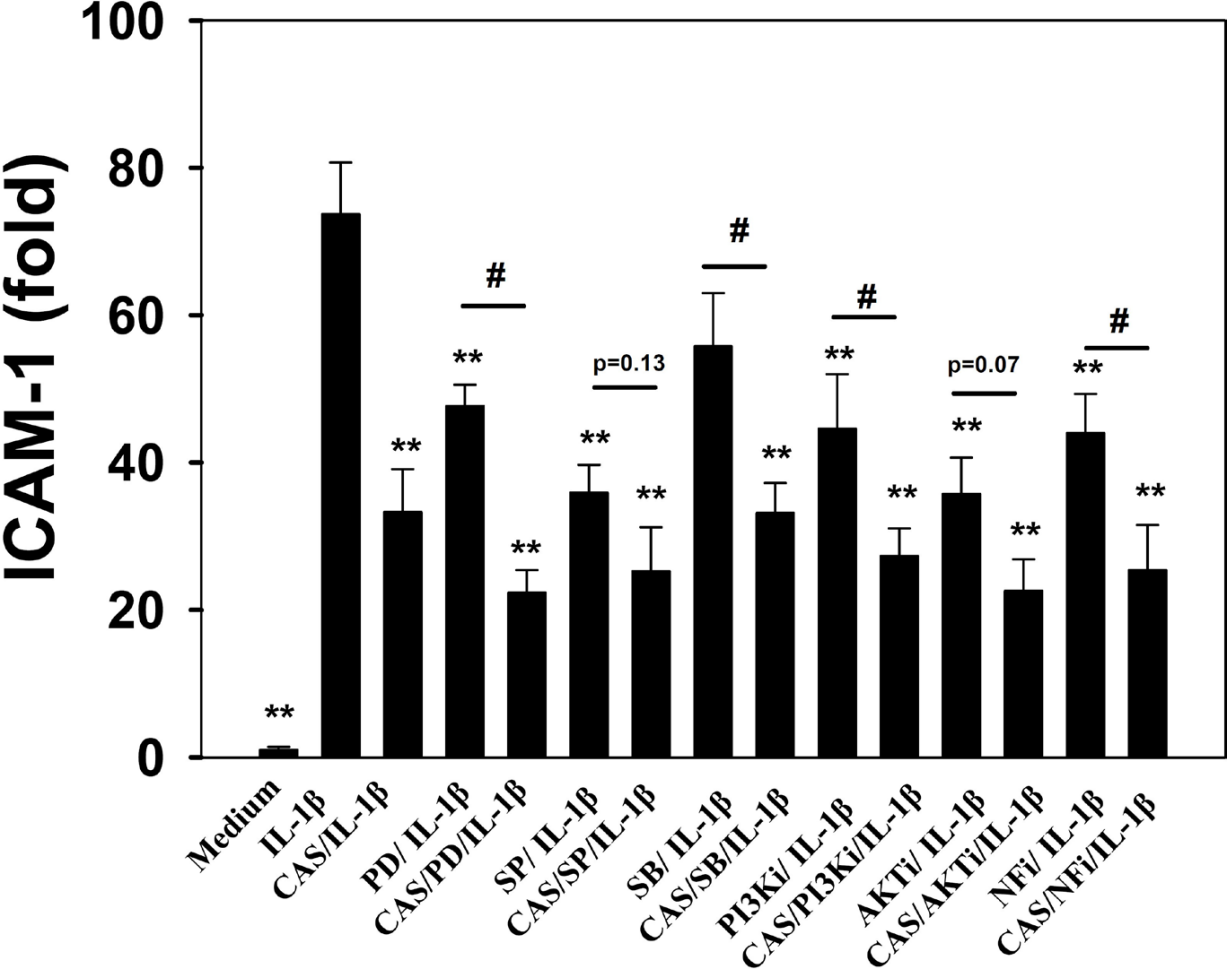
Inhibitory effects of MAPK, PI3K, Akt, and NF-κB inhibitors or casticin (CAS) on IL-1β–induced ICAM-1 protein expression in A549 cells A549 cells were pretreated with ERK1/2 inhibitors (PD: 10 μM PD98059), p38 inhibitors (SB: 10 μM SB203580), JNK inhibitors (SP: 10 μM SP600165), PI3K inhibitor (PI3Ki: 10 μM AS604850), AKT inhibitor (AKTi: 10 μM Akt1/2 kinase inhibitor), and NF-κB inhibitor (NFi: 10 μM BAY11-7085) with or without 20 μM CAS for 1 h, followed by IL-1β stimulation for 4 h. ICAM-1 gene expression was detected by real-time PCR. The presented data are mean±SEM; ^*^p < 0.05, ^**^p < 0.01, compared with the IL-1β–treated group. ^#^*p* < 0.05, ^##^*p* < 0.01, compared with the inhibitor / IL-1β group.

Casticin suppressed proinflammatory cytokine, chemokine, and ICAM-1 expression. Thus, we also evaluated whether casticin suppressed the NF-κB, MAPK, and Akt/PI3K pathways in IL-1β–induced A549 cells. The results showed that casticin significantly suppressed phosphorylation of JNK, p38, and ERK1/2 (Figure 6A, 6B). In addition, casticin decreased Akt and PI3K phosphorylation compared with IL-1β–activated A549 cells (Figure 6C, 6D). Furthermore, it significantly suppressed IκB-α phosphorylation, increased IκB-α degradation, and reduced p65 translocation into the nucleus compared with IL-1β–induced A549 cells (Figure 7A–7C). The NF-κB promoter assay results showed that casticin significantly reduced luciferase activity compared with IL-1β–stimulated A549 cells (Figure 7D). When PI3K, AKT, MAPK, and NF-κB inhibitors were used to evaluate the inhibitory effect of casticin on ICAM-1 expression with real-time PCR, we found that it could also enhance ERK, p38, PI3K, and NF-κB inhibitors to suppress ICAM-1 gene expression (Figure 8).

## DISCUSSION

The fruit of *V. rotundifolia* is a medical herb commonly used to treat inflammation, colds, bronchitis, and headaches in traditional medicine [32]. Casticin is a flavonoid isolated from *V. rotundifolia* [29]. Recent studies have found that casticin has potential anticancer effects by inducing apoptosis of cancer cells, including human lung cancer, hepatocellular carcinoma, gastric cancer, glioma, and cervical cancer cells [32–36]. Casticin also can suppress ICAM-1 expression, decrease eosinophil migration into lung epithelial cells, and attenuate the acute lung inflammatory response in cigarette smoke–induced lung disease in mice [28, 37]. Previously, we found that casticin has an anti-inflammatory effect of suppressing proinflammatory cytokines by blocking the MAPK, NF-κB, and Akt signaling pathways in LPS-stimulated macrophages [30]. In the present study, casticin decreased ICAM-1 expression and suppressed the ability of monocyte THP-1 cells to adhere to IL-1β–stimulated A549 cells. Casticin reduced levels of COX-2 and PGE_2_ and decreased proinflammatory cytokine and chemokine protein and gene expression. Casticin also significantly reduced inflammatory process–associated signaling pathways, including phosphorylation of Akt-PI3K and MAPKs and NF-κB p65 translocation into the nucleus. Thus, we suggest that casticin may ameliorate the inflammatory effect in IL-1β–stimulated lung epithelial cells.

IL-1β, is belonged to inducible proinflammatory cytokine, did not generally secrete in healthy tissue [7, 31]. In the development of chronic or acute inflammatory diseases, the tissue expressed high amount of IL-1β to cause cell and tissue damage [1, 15]. When Gram-negative bacteria infect the lungs, they release LPS, which stimulates macrophage activation and excessive secretion of inflammatory cytokines IL-1β, TNF-α, and IL-6 [20, 38]. Those cytokines irritate and destroy lung epithelial cells, leading to airway inflammation related to bacteria pneumonia or lung injury [10, 20]. Recent studies have found that IL-1β is the major cytokine inducing lung inflammation in acute respiratory distress syndrome, which involves a decrease in blood oxygen that is sufficient to cause hypoxic damage [39]. Idiopathic pulmonary fibrosis is a progressive lung disease involving IL-1β–stimulated lung epithelial cells in which epithelial repair processes, lung inflammation, and fibroproliferation are triggered [40]. IL-1RA can bind the IL-1 receptor to block the activity of the IL-1β signaling pathway [41] and ameliorate eosinophil infiltration, airway hyperresponsiveness, Th2 cytokine levels in bronchoalveolar lavage fluid, and airway remodeling in asthmatic mice [42]. Additionally, in idiopathic pulmonary fibrosis, the lung expresses high levels of IL-1β, IL-17A, and IL-23. Treatment with IL-1RA suppresses IL-23 and IL-17A production and attenuates the symptoms of idiopathic pulmonary fibrosis [40]. In this study, we showed that casticin more significantly suppresses ICAM-1 and MUC5AC gene expression when IL-1β–stimulated A549 cells are treated with IL-1RA. Hence, casticin exerts an inflammatory effect by blocking the IL-1β pathway in lung epithelial cells. Furthermore, previous study found that H460 cells treated with epidermal growth factor, and H460 cells could significantly increase the levels of IL-6 and IL-6 receptor [43]. Hence, suppressed interleukin-6 receptor could reduce the proliferation of H460 cells for lung cancer therapeutics [43]. However, we found that IL-1β-stimulated H460 did not significantly increase IL-6 and IL-8 productions. We thought that H460 did not have IL-1 receptors to induce the IL-1β signaling pathway for increasing IL-8 and IL-6 expression.

ICAM-1 is a cell adhesion molecule, and inflammatory lung epithelial cells highly express ICAM-1, leading to greater adherence of neutrophils or monocytes that infiltrate the lung tissue [44]. These neutrophils or monocytes release excessive proinflammatory cytokines that aggravate damage and inflammation [41, 45]. In the current study, we confirm that casticin reduces ICAM-1 gene expression and protein production and suppresses the promoter activity of ICAM-1 in inflammatory A549 cells. However, casticin also did not significantly modulate VCAM-1 gene expression in IL-1β–stimulated A549 human lung epithelial cells (data not shown). We also found that casticin reduced THP-1 adhesion to A549 cells and blocked entry of more inflammatory cells. Therefore, we thought that casticin mainly inhibited ICAM-1 expression to block inflammatory cells into the lung tissus.

Casticin could inhibite NF-κB, PI3K/Akt, and MAPK activity. PI3K/Akt signaling can induce NF-κB activity and translocation into the nucleus. IL-1β–stimulated A549 cells also use MAPK signaling to regulate NF-kB activation for ICAM-1 expression [44, 46]. IL-1β–stimulated A549 cells treated with NF-κB, PI3K/Akt, and MAPK inhibitors along with casticin and found more significantly decreased ICAM-1 expression compared with IL-1β–stimulated A549 cells. JNK and Akt inhibitor co-cultured with casticin did not significantly suppress ICAM-1 expression, however.

Epithelial cells of airways and lung tissue secrete mucus in response to toxic particles and microbes to protect against lung damage [1, 47]. Inhaled smoke or nicotine is associated with many lung diseases, including emphysema, chronic bronchitis, and COPD, and inhaled allergens may cause asthma [3, 48]. Emphysema is a chronic pulmonary disease that causes difficulty breathing by alveolar overinflation [49]. In chronic bronchitis, tobacco smoke can lead to inflammation of airways for reduced air flow and caused coughing [1]. These lung diseases involve relatively more mucin secretion in the epithelial layers of airways. Cilia are the principal cells on the surface epithelium of intrapulmonary airways for excluding inhaled microbes and dust [50, 51]. Epithelial cells also release more antimicrobial molecules, including IgA and lysozyme, against microbial infections [1]. However, when inflammatory lung epithelial cells secrete excessive mucus, the airway can be obstructed, causing difficulty breathing and suffocation [52]. Casticin has an anti-inflammatory effect and significantly suppressed MUC5AC expression in A549 cells in the current work.

The COX-2 inhibitor meloxicam suppresses arachidonic acid conversion into PGE_2_ [53]. We previously found that casticin has an anti-inflammatory effect by decreasing COX-2 and PGE_2_ production in LPS-stimulated macrophages [30]. In the current work, casticin also significantly reduced COX-2 and PGE_2_ production in IL-1β–activated A549 cells.

Neutrophils are essential for removing bacterial infections in acute inflammatory lung disease [31]. Lung epithelial cells of patients release IL-8 to attract more neutrophils against microbe infection [54], and inflammatory epithelial cells secrete more IL-1β, IL-6, TNF-α, CCL5, MCP-1, and IL-17 to activate the inflammatory warning signal [51]. However, those inflammatory responses of the airways also can cause lung injury and physical discomfort. Casticin significantly reduces inflammatory cytokine and chemokine production in inhibiting the inflammatory response of lung epithelial cells. A previous study found that A549 cells stimulated with TNF-α/IL-4/IL-1β release eotaxin and that casticin suppresses eotaxin production [28]. Interestingly, we found that casticin did not decrease eotaxin (e.g., CCL11 and CCL24 expression) in IL-1β–stimulated A549 cells. Our previous study found that human bronchial epithelial (BEAS-2B) cells induce eotaxin expression by stimulation with TNF-α/IL-4 [45]. Thus, we speculate that IL-1β–stimulated A549 cells in the current work did not significantly release more eotaxin to attract eosinophil migration. CCL17, is a thymus and activation-regulated chemokine, associated with chronic pulmonary inflammation in asthma [55]. A previous study found that CCL17 could selectively attract Th2 cells activation in asthmatic mice [56]. Casticin did not modulate CCL17 gene expression in IL-1β–stimulated A549 cells. We speculated that casticin did not suppress CCL17 production for blocked more Th2 cells infiltration into the lung tissue. Furthermore, EGFR could enhance inflammatory responses in A549 cells [57]. C/EBP transcription factors also modulate inflammatory gene expression in lung diseases [58], and here we found that casticin represses C/EBPβ and EGFR inflammatory gene expression in A549 cells.

**Figure 9:**
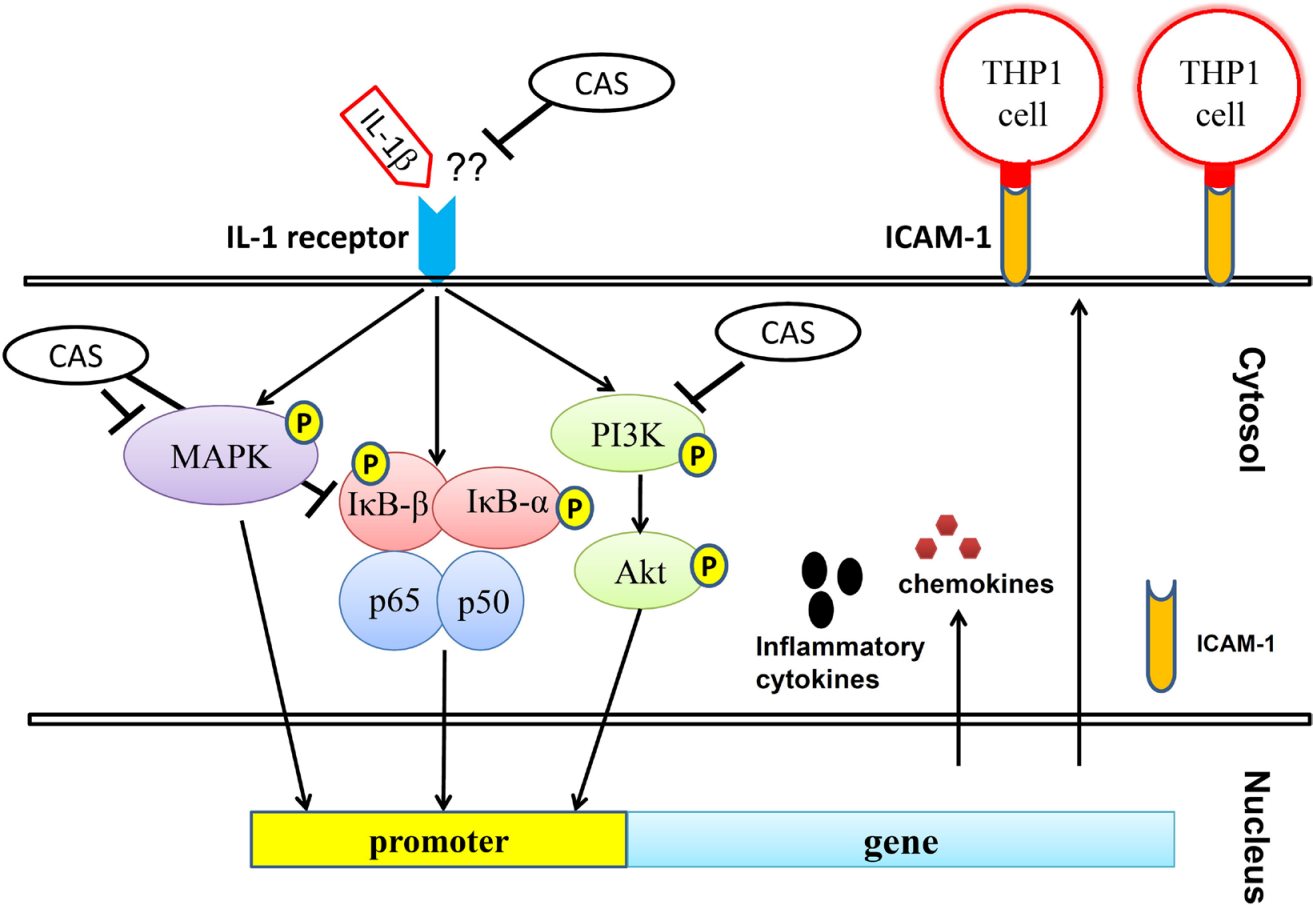
Model explaining the mechanism for the anti-inflammatory effects of casticin (CAS) in IL-1β–induced A549 cells

Airways affected by airborne microbes, smoke, or air pollution activate lung epithelial cells and macrophages to secrete a number of proinflammatory cytokines and mediators that contribute to the pathophysiology of pneumonia [10, 59]. Casticin decreased proinflammatory cytokine, chemokines, ICAM-1, and MUC5AC expression by blocking activation of the NF-κB, PI3K-Akt, and MAPK signaling pathways in IL-1β–activated human lung epithelial cells (Figure 9). Casticin also suppressed leukocyte adherence to lung epithelial cells by reducing ICAM-1 expression via inhibition of the IL-1β pathway. This compound holds potential as a natural anti-inflammatory drug that could ameliorate inflammatory lung disease.

## MATERIALS AND METHODS

### Materials

Casticin (≥ 98% purity by HPLC) was purchased from Sigma-Aldrich (Sigma, St. Louis, MO, USA) and dissolved in DMSO. The concentration of the stock solution was 100 mM. DMSO was ≤ 0.1% in culture medium, as previously described [30].

### Cell line and culture medium

Human lung epithelial A549 cells and H460 cells were obtained from the Bioresource Collection and Research Center (BCRC, Taiwan). Cells were routinely cultured in Dulbecco's modified Eagle's medium (Invitrogen-Gibco^TM^, Paisley, Scotland) supplemented with 10% fetal bovine serum, 100 U/ml penicillin, and 100 μg/ml streptomycin. Cells were incubated in 5% CO_2_ humidified air at 37°C and subcultured twice each week.

### Cell viability assay

Cell viability was measured using the MTT assay, as previously described [60]. In brief, A549 and H460 cells were seeded in 96-well plates and treated with various concentrations of casticin in each well, and the plates were incubated at 37°C for 24 h. An MTT working solution (5 mg/ml) was added to the plates for 4 h at 37°C, and then formazan crystals were dissolved using isopropanol. Finally, the absorbance of each well at 570 nm was measured with a microplate reader (Multiskan FC, Thermo, Waltham, MA, USA). Three independent experiments were carried out for each of the different treatments.

### RNA isolation and real-time PCR for gene expression

Total RNA for detection of A549-related cytokines, chemokines, and ICAM-1 was extracted after stimulation and treatment with casticin. cDNA was synthesized using a cDNA synthesis kit (Life Technologies), and gene expression was measured by incorporation of fluorescent SYBR Green using a spectrofluorometric thermal cycler (iCycler; Bio-Rad Laboratories, Hercules, CA, USA). Specific primers were designed as shown in Table 1. PI3K inhibitor (AS604850) and AKT inhibitor (Akt1/2 kinase inhibitor) (Sigma), NF-κB inhibitor (BAY11-7085), and MAPK inhibitors (JNK inhibitor SP600125, ERK inhibitor PD98059, and p38 inhibitor SB203580) (Enzo Life Sciences, Inc., Farmingdale, NY, USA) were cultured with casticin for detection of ICAM-1 gene expression.

**Table 1:** Primers used in real-time PCR analyses of mRNA expression

Gene	Primer Forward	Primer Reverse
CCL5	CGAAGGAACCGCCAAGTGT	AGGACTAGAGCAAGCAATGAC
CCL11	GGCTTCATGTAGTTCCAGAT	CCATTGTGTTCCTCAATAATCC
CCL17	GCCTTGAGAGGTCTTGAAGC	TCACTGTGGCTCTTCTTCGT
CCL24	AGGCAGTGAGAACCAAGT	GCGTCAATACCTATGTCCAA
CCL26	AGACCTGCTGCTTCCAATACA	GGGTACAGACTTTCTTGCCTCT
COX-2	ACCAGCAGTTCCAGTATCAGA	CAGGAGGATGGAGTTGTTGTAG
IL-1β	CACTACAGGCTCCGAGATGA	CGTTGCTTGGTTCTCCTTGT
IL-4	TCCGTGCTTGAAGAAGAACTC	GTGATGTGGACTTGGACTCATT
IL-6	AGGACCAAGACCATCCAATTCA	GCTTAGGCATAACGCACTAGG
IL-8	GCAGAGGGTTGTGGAGAAGT	TGGCATCTTCACTGATTCTTGG
IL-13	GCTCCAGCATTGAAGCAGTG	CGTGGCAGACAGGAGTGTT
IL-17A	CACCTCACCTTGGAATCTC	GGATCTCTTGCTGGATGG
IL-17F	ACACAGGCATACACAGGAAGA	CCAATATCGACAGCAGCAAGTA
MCP-1	TTCCACAACCACCTCAAGCA	TTAAGGCATCACAGTCCGAGTC
TNF-α	GCACCACCATCAAGGACTC	AGGCAACCTGACCACTCTC
ICAM-1	AACAGAATGGTAGACAGCAT	TCCACCGAGTCCTCTTAG
MUC5AC	GTGTCCACTGTGTCCTCCTC	GGCTCGGTCGGTCTTATTGT
C/EBPα	GACTTGGTGCGTCTAAGATGAG	TAGGCATTGGAGCGGTGAG
C/EBPβ	GTCCAAACCAACCGCACAT	CAGAGGGAGAAGCAGAGAGTT
EGFR	GCCAAGGCACGAGTAACAAG	CCAAGGACCACCTCACAGTT
β-actin	AAGACCTCTATGCCAACACAGT	AGCCAGAGCAGTAATCTCCTTC

### ELISAs for chemokines, cytokines, and PGE_2_ production

A549 and H460 cells were pretreated with or without casticin in 24-well plates for 1 h, then stimulated with IL-1β (1 ng/ml) and cultured for 24 h to assay chemokine, cytokine, and PGE_2_ production using specific ELISA kits (R&D Systems, Minneapolis, MN, USA). The absorbance of each well at 450 nm was measured with a microplate reader (Multiskan FC, Thermo, Waltham, MA, USA). Three independent experiments were carried out for each of the different treatments.

### Preparation of total and nuclear proteins

A549 cells were seeded in 6-well plates and treated with casticin for 1 h. Then, cells were stimulated with IL-1β (1 ng/ml) for 30 min to evaluate protein phosphorylation or for 24 h to detect total protein expression. Proteins were extracted using protein lysis buffer containing protease and phosphatase inhibitors (Sigma). Additionally, nuclear proteins were isolated using the NE-PER^®^ nuclear and cytoplasmic extraction reagent kits (Pierce, Rockford, IL, USA), and proteins were quantified with the BCA protein assay kit (Pierce).

### Western blotting analysis

Proteins were separated on SDS polyacrylamide gels and transferred onto PVDF membranes (Millipore, Billerica, MA, USA). The membranes were incubated overnight at 4°C with specific primary antibodies, including Akt, phosphorylated-Akt, COX-2, IκB-α, phosphorylated-IκB-α, p65, and Lamin B1 (Santa Cruz, CA, USA); ERK1/2, phosphorylated-ERK 1/2, p38, phosphorylated-p38, JNK, and phosphorylated-JNK (Millipore); PI3K and phosphorylated-PI3K (Cell Signaling Technology, Inc., MA, USA); and ICAM-1 and β-actin (Sigma). The membrane was washed and incubated with secondary antibodies for 1 h. Finally, specific proteins were detected with Luminol/Enhancer Solution (Millipore) using the BioSpectrum 600 system (UVP, Upland, CA, USA).

### Transient transfection and luciferase activity assay

NF-κB activity was evaluated using transfected pNFκB-Luc plasmid (Stratagene, CA, USA), as previously described [45]. Additionally, we constructed the pICAM1-Luc plasmid, in which the region of the human ICAM-1 promoter (1.2-kb) in a luciferase reporter vector pMuc-Luc was amplified from A549 cells using PCR, and the DNA sequence was confirmed by dideoxynucleotide sequencing. A549 cells were transfected with 1 μg pNFκB-Luc or pICAM1-Luc plasmid using Lipofectamine 2000 (Life Technologies, Carlsbad, CA, USA) for 24 h. The cells were treated with casticin for 1 h, and then IL-1β was added for 4 h. Luciferase activity was measured using a luciferase reporter assay kit (GeneDireX, Brussels, Belgium) and determined with a Multi-Mode microplate reader (BioTek Synergy HT, Bedfordshire, United Kingdom).

### Cell–cell adhesion assay

A549 cells were pretreated with casticin and incubated with IL-1β for 24 h. Then, they were treated with calcein AM (Sigma) from human monocytic THP-1 cells, which were co-cultured with A549 cells for 1 h. Cells were observed using fluorescence microscopy (Olympus, Tokyo, Japan).

### Statistical analysis

All experimental data were analyzed by one-way analysis of variance and post hoc analyses with Dunnett's test. Data are presented as the mean ± SEM of at least three independent experiments, and statistical significance was set at *p* < 0.05.

## SUPPLEMENTARY MATERIALS FIGURES



## Abbreviations

COX-2: cyclooxygenase 2
EGFR: epidermal growth factor receptor
ICAM-1: intercellular adhesion molecule 1
IL-1β: interleukin-1β
MAPK: mitogen-activated protein kinase
MUC5AC: mucin 5AC
PI3K: phosphatidylinositol 3-kinase
NF-κB: nuclear transcription factor kappa-B
PGE_2:_: prostaglandin E2
TNF-α: tumor necrosis factor α
