# Supplementary material for: Casticin inhibits interleukin-1β–induced ICAM-1 and MUC5AC expression by blocking NF-κB, PI3K-Akt, and MAPK signaling in human lung epithelial cells
Source: Oncotarget. 2017 Sep 15;8(60):101175–88. doi: 10.18632/oncotarget.20933 (PMC5731865; doi:10.18632/oncotarget.20933)
Supplement: Supplementary file 1 [file oncotarget-08-101175-s001.pdf]

Casticin inhibits interleukin-1β–induced ICAM-1 and MUC5AC expression by blocking NF-κB, PI3K-Akt, and MAPK signaling in human lung epithelial cells

SUPPLEMENTARY MATERIALS

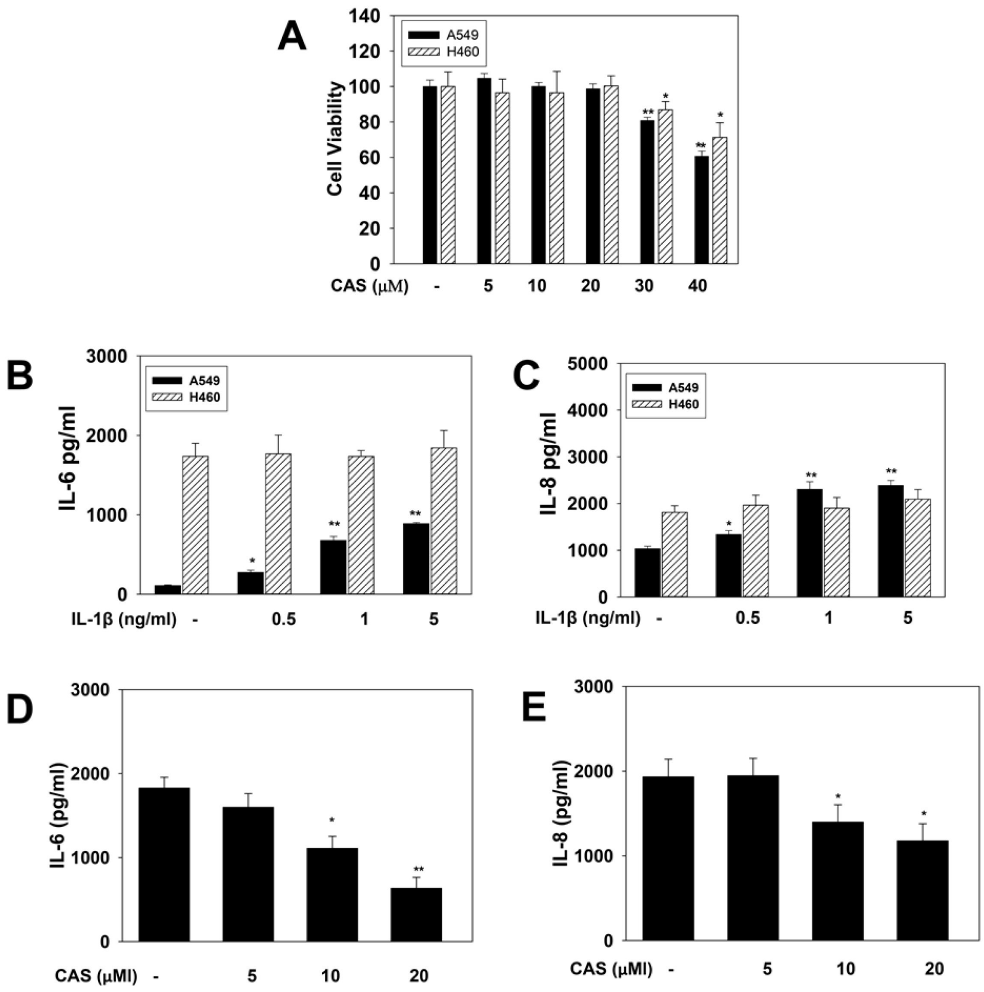

Supplementary Figure 1: A549 and H460 cells were treated with various concentrations of casticin, and cell viability was measured using the MTT assay. (**A**) Next, A549 and H460 cells were treated with various doses of IL-1β (0.5–5 ng/ml) for 24 h. IL-6 (**B**) and IL-8 (**C**) levels were measured by ELISA. Furthermore, H460 cells were treated with CAS (0–20 μM) for 24 h. IL-6 (**D**) and IL-8 (**E**) levels were measured by ELISA. The presented data are mean ± SEM; $^{*}p < 0.05$, $^{**}p < 0.01$, compared with the untreated cells.
